# MicroRNA expression profile associated with response to neoadjuvant chemoradiotherapy in locally advanced rectal cancer patients

**DOI:** 10.1186/1748-717X-7-195

**Published:** 2012-11-20

**Authors:** Marek Svoboda, Jiri Sana, Pavel Fabian, Ilona Kocakova, Jana Gombosova, Jana Nekvindova, Lenka Radova, Rostislav Vyzula, Ondrej Slaby

**Affiliations:** 1 Department of Comprehensive Cancer Care, Masaryk Memorial Cancer Institute, Zluty kopec 7, Brno, 656 53, Czech Republic; 2 Department of Cancer Epidemiology and Genetics, Masaryk Memorial Cancer Institute, Brno, Czech Republic; 3 Department of Oncological and Experimental Pathology, Masaryk Memorial Cancer Institute, Brno, Czech Republic; 4 Department of Radiation Oncology, Masaryk Memorial Cancer Institute, Zluty kopec 7, Brno, Czech Republic; 5 Central European Institute of Technology, Masaryk University, Kamenice 5, Brno, Czech Republic; 6 Institute of Clinical Biochemistry and Diagnostics, Faculty of Medicine and Faculty Hospital in Hradec Kralove, Charles University, Hradec Kralove, Czech Republic; 7 Laboratory of Experimental Medicine, Institute of Molecular and Translational Medicine, Faculty of Medicine and Dentistry, Palacky University and Palacky University affiliated Hospital, Olomouc, Czech Republic

**Keywords:** Rectal cancer, microRNA, Prediction, Neoadjuvant chemoradiotherapy, Radiation, Capecitabine, 5-fluorouracil, Thymidylate synthetase

## Abstract

**Background:**

Rectal cancer accounts for approximately one third of all colorectal cancers (CRC), which belong among leading causes of cancer deaths worldwide. Standard treatment for locally advanced rectal cancer (cT3/4 and/or cN+) includes neoadjuvant chemoradiotherapy with fluoropyrimidines (capecitabine or 5-fluorouracil) followed by radical surgical resection. Unfortunately, a significant proportion of tumors do not respond enough to the neoadjuvant treatment and these patients are at risk of relapse. MicroRNAs (miRNAs) are small non-coding RNAs playing significant roles in the pathogenesis of many cancers including rectal cancer. MiRNAs could present the new predictive biomarkers for rectal cancer patients.

**Methods:**

We selected 20 patients who underwent neoadjuvant chemoradiotherapy for advanced rectal cancer and whose tumors were classified as most sensitive or resistant to the treatment. These two groups were compared using large-scale miRNA expression profiling.

**Results:**

Expression levels of 8 miRNAs significantly differed between two groups. MiR-215, miR-190b and miR-29b-2* have been overexpressed in non-responders, and let-7e, miR-196b, miR-450a, miR-450b-5p and miR-99a* have shown higher expression levels in responders. Using these miRNAs 9 of 10 responders and 9 of 10 non-responders (p < 0.05) have been correctly classified.

**Conclusions:**

Our pilot study suggests that miRNAs are part of the mechanisms that are involved in response of rectal cancer to the chemoradiotherapy and that miRNAs may be promising predictive biomarkers for such patients. In most miRNAs we identified (miR-215, miR-99a*, miR-196b, miR-450b-5p and let-7e), the connection between their expression and radioresistance or chemoresistance to inhibitors of thymidylate synthetase was already established.

## Introduction

Carcinomas of the colon and rectum (colorectal cancer, CRC) are among leading causes of cancer-related deaths worldwide and in Czech Republic, the incidence of CRC is one of the highest in the world [1,2]. Cancer of the rectum and rectosigmoideal junction (hereinafter referred to as “rectal tumors”) make up 30% to 40% of all CRC [3,4] and adenocarcinomas represent the absolute majority of them [4]. About 45% of rectal tumors is diagnosed as locally advanced rectal adenocarcinoma (LARA) in Czech Republic [3]. According to TNM classification, these are tumors in clinical stage II. and III., defined as cT3 or cT4, and/or tumors in which the regional lymphatic nodes are affected (cN1 or cN2) [5]. Compared to colon tumors, LARA are more likely, after surgical treatment, to relapse locally, metastasize to lungs and often lead to a serious decline in quality of patient’s life [6-8]. To achieve a better local control of the disease and, possibly, to increase the probability of a radical surgery with preservation of the anal sphincter and avoidance of a permanent colostomy, LARA treatment is based most often on application of neoadjuvant chemoradiotherapy with fluoropyrimidines followed by surgical treatment and, eventually, adjuvant chemotherapy [9-13].

The neoadjuvant chemoradiotherapy brings a significant regression in more than 2/3 of tumors (TRG1, TRG2, and most of tumors evaluated as TRG3 according to Mandard’s classification; TRG – tumor regression grade) and in approximately 15 – 20% of patients the complete eradication of tumor is achieved [11-13], called a pathological complete response (pCR; ypT0 in TNM classification, TRG1 in Mandard’s classification) [14]. Several retrospective analyses suggest that the tumor stage after neoadjuvant treatment has a significant prognostic impact on a disease-free (DFS) and overall survival (OS) [11-13]. There is, nevertheless, a significant subgroup of rectal cancer patients (approx. 20%) having poor response to the neoadjuvant therapy (TRG5, TRG4 and a part of TRG3 classified tumors) [14-16]. Therefore, the ability to predict response for neoadjuvant chemoradiotherapy may allow individualization and more rational selection of patients that will most likely benefit from this therapy.

MicroRNAs (miRNAs) are highly conserved, small, non-coding RNAs, 18–25 nucleotides in length, that act as post-transcriptional regulators of gene expression by silencing their mRNA targets. Recent studies showed that miRNAs regulate a significant number of oncogenes, tumor suppressor genes, and genes associated with the invasion, dissemination, and therapy resistance of many tumors [17]. In colorectal cancer, polymorphisms within miRNAs binding regions have been described as new risk factors. Several genome-wide profiling studies have identified miRNAs deregulated in CRC tissue. A number of experimental studies on these miRNAs revealed insight into miRNA-mediated regulatory links to well-known oncogenic and tumor suppressor signaling pathways [17]. Several investigations have also described the ability of specific miRNA expression profiles to predict prognosis and therapy response in CRC patients [17-19].

The aim of this study was to analyse global miRNA expression profiles in the clinical samples of rectal tumors to identify miRNA signatures specific for responders and non-responders to neoadjuvant chemoradiotherapy in patients with LARA.

## Material and methods

### Patients

This retrospective study included 20 patients with previously untreated and histologically confirmed locally advanced rectal adenocarcinoma (LARA). All patients underwent neoadjuvant chemoradiotherapy based on concurrent application of radiotherapy (45 Gy to pelvis plus 5.6 Gy boost to tumor; 1,8 Gy/day, 5 days/week with all fields being treated daily) and chemotherapy (capecitabine 825 mg/m2, per os, twice a day daily, or 5-fluorouracil as a continuous i.v. infusion at a daily dose 225 mg/m2, both drugs were administered throughout the radiotherapy) which was followed by surgical treatment of the tumor and, eventually, adjuvant chemotherapy by the same cytostatic that was used in neoadjuvant therapy. The dose of radiotherapy or chemotherapy was not reduced in any of the patients. Surgery was scheduled 6 weeks after completion of chemoradiotherapy. Techniques of surgery were standardized and we are basically looking at three types of surgery (APR – abdominoperineal resection, LAR – low anterior resection, uLAR – ultra low anterior resection); TME (total mesorectal excision) was always performed. The response of the tumor to neoadjuvant therapy was evaluated in several ways. TNM classification was used for clinical purposes (5). For our project, evaluation of tumor regression was done in two ways: 1. using a grading system adapted from Mandard et al. (14), and 2. establishing an average and a maximal percentual representation of residual cancer cells in the cell population detected in 10 examined slices of formalin fixed and paraffin embedded primary tumors. Of these 20 patients, 10 patients responded and 10 patients did not respond to the neoadjuvant chemoradiotherapy. Patients that responded to the therapy (the “responders”) were those whose tumor regression after the therapy reached the classification TRG1 or TRG2. In tumors classified as TRG2, the average of residual tumor cells could not exceed 10% of the entire cell population detected in the examined slices and/or 50% in at least one of the examined slices. Patients that did not respond to the therapy (the “non-responders”) were those whose tumors showed after the neoadjuvant therapy no regression (TRG5) or just partial regression in cancer cell population (TRG4 and TRG3). Tumors evaluated as TRG3 were marked as non-responders only when maximal number of residual cancer cells reached 50% in at least one of the examined slices. Informed consent was obtained from all patients, and the local Ethical Board approved the study protocol. Detailed characteristic of patients and their tumors is summarized in Table 1.

**Table 1:** Patient characteristics

	**Patient**	**Gender**	**Age**	**Clinical stage**	**AC [cm]**	**Chemotherapy**	**CEA**	**CA 19-9**	**Type of surgery**	**Histology**	**Grade**	**L/V/P**	**cT**	**cN**	**ypT**	**ypN**	**TRG**	**Residual cancer cells Avg/Max %**	**Local relaps**	**Distant relaps**
**Responders**	**01**	M	30	IIIA	12	Xel	N	N	LAR	A	1	0	1	1	1	0	2	1/5	0	0
	**02**	F	62	IIIA	13	Xel	N	N	LAR	A	2	0	x	1	1	1	2	2,5/5	0	0
	**03**	F	67	IIA	3,5	Xel	N	N	APR	A	2	0	3	0	1	0	2	7,5/15	0	0
	**04**	M	43	IIIA	9	Xel	N	N	uLAR	A	1	0	2	1	0	0	1	0/0	0	0
	**05**	M	40	IIIB	5	Xel	E	N	APR	A	2	0	3	1	3	1	2	1/5	0	0
	**06**	M	73	IIA	6,5	Xel	N	E	uLAR	A	2	0	3	0	0	0	1	0/0	0	0
	**07**	M	68	IIIC	6	Xel	E	E	APR	A	2	0	3	2	0	0	1	0/0	0	0
	**08**	M	58	IIIB	13	Xel	N	N	APR	A	1	0	3	1	3	0	2	2,5/40	0	Y
	**09**	M	68	IIA	4,5	5FU	N	N	APR	A	2	0	3	0	0	0	1	0/0	0	0
	**10**	M	43	IIA	4	Xel	N	N	uLAR	MA	2	0	3	0	0	0	1	0/0	0	0
**Non-responders**	**11**	M	63	IIIB	7	Xel	E	N	uLAR	A	2	0	3	1	2	0	4	15/50	0	0
	**12**	M	59	IIIB	9	Xel	E	N	LAR	A	2	0	3	1	3	0	4	40/60	0	0
	**13**	M	45	IIIC	4	Xel	N	N	APR	A	2	1P	3	2	3	1	3	7,5/50	Y	Y
	**14**	F	64	IIIC	11	Xel	N	N	LAR	A	2	0	3	2	2	0	4	50/70	0	0
	**15**	F	60	IIIA	9	Xel	N	N	LAR	A	1	0	2	1	1	0	4	70/80	0	0
	**16**	F	69	IIIC	8	Xel	N	N	LAR	A	1	0	3	2	3	1	4	25/70	0	0
	**17**	M	32	IIIC	10	Xel	N	N	LAR	A	2	0	2	2	3	0	4	55/75	0	0
	**18**	M	53	IIIC	4	Xel	N	N	APR	A	3	0	3	2	2	0	3	40/60	0	0
	**19**	M	63	IV*	7	5FU	E	N	APR	MA	2	0	3	2	3	2	4	60/80	0	Y
	**20**	F	52	IIA	8	Xel	N	N	LAR	A	2	0	3	0	2	1	3	60/70	0	0

N – Normal; E – Elevated; LAR – Low Anterior Resection; uLAR – Ultra (extended) Low Anterior Resection; APR –Abdominoperineal Resection; A – Adenocarcinoma; MA – Mucinous Adenocarcinoma; L/V/P – Lymphatic/Vascular/Perineural Invasion; 1P – Positive Perineural Invasion; TRG – Tumor Regression Score according to Mandard et al. (10); CEA – Carcinoembryonic antigen; CA 19–9 – cancer antigen 19–9; Clinical stage – according to TNM /UICC cancer staging classification, 6th Edition (ref. 5); cT/cN – clinical staging of primary tumor (T) / regional lymphatic nodes (N); ypT/ypN – histopathological staging of primary tumor and regional lymphatic nodes after neoadjuvant treatment; Residual cancer cells (Avg/Max %) – a proportion of cancer cells in all paraffin embedded tisue slices of primary tumor histologicaly examined after neoadjuvant treatment: Average (%) / Maximum (%) of cancer cells presented in examined slices; Xel – Xeloda (capecitabine); 5FU – 5-fluorouracil; M – male; F – female; AC – distance between the anocutaneous line and distal (aboral) margin of the tumor in cm; Y – yes; * in such patient a radical metastasectomy of solitary liver metastasis was first performed, followed by neoadjuvantchemoradiotherapy for locally advanced rectal cancer.

### Tissue sample preparation and miRNA isolation

Bioptic samples of untreated primary tumors were immediately stored in liquid nitrogen until RNA extraction. All analyzed tissues were homogenized (Retsch MM301) and total RNA enriched for small RNAs was isolated using mirVana miRNA Isolation Kit (Ambion, USA). Nucleic acid concentration and purity were controlled by UV spectrophotometry (A260/A280 > 2.0; A260/A230 > 1.8) using Nanodrop ND-1000 (Thermo Fisher Scientific, USA).

### Large-scale miRNA expression profiling

We performed TaqMan Low Density Arrays (TLDA) analysis to identify profile of differentially expressed miRNAs between the two sets of biopsy samples (10 responders and 10 non-responders to neoadjuvant chemoradiotherapy for rectal cancer). In brief, 350 ng of total RNA was reverse-transcribed into cDNA by the TaqMan MicroRNA Reverse Transcription Kit and Megaplex RT set pool A and B version 2.0 (Applied Biosystems, USA). The RT product was loaded into TaqMan Array Human MicroRNA A+B Cards Set v2.0 (Applied Biosystems, USA) enabling simultaneous quantitation of 667 human miRNAs. TaqMan MicroRNA Assays and analysis were performed on the ABI 7900HT Instrument (Applied Biosystems, USA). All reactions were performed according to the standard manufacturers’ protocols. Quantitative miRNAs expression data were acquired and normalized by use of ABI 7900HT SDS software (Applied Biosystems, USA).

### Statistical methods

The obtained primary data were analysed using the SDS 2.0.1 software and RQ Manager 1.2 (Applied Biosystems, USA) (settings: automatic baseline, threshold 0.2). RNU48 has been used as reference gene for normalization of miRNAs expression levels. The relative expression levels of target miRNAs were determined by the equation 2^−ΔCT^, in which ΔCT were calculated as follows: ΔCT = CT miR-of-interest − CT RNU48. Normalized expression data were statistically evaluated in the environment of statistical language R by use of Bioconductor package and LIMMA approach combined with hierarchical clustering (HCL) [20]. Putative miRNAs’ targets were predicted using miRWalk database and miRanda algorithm [21].

## Results

**Figure 1:**
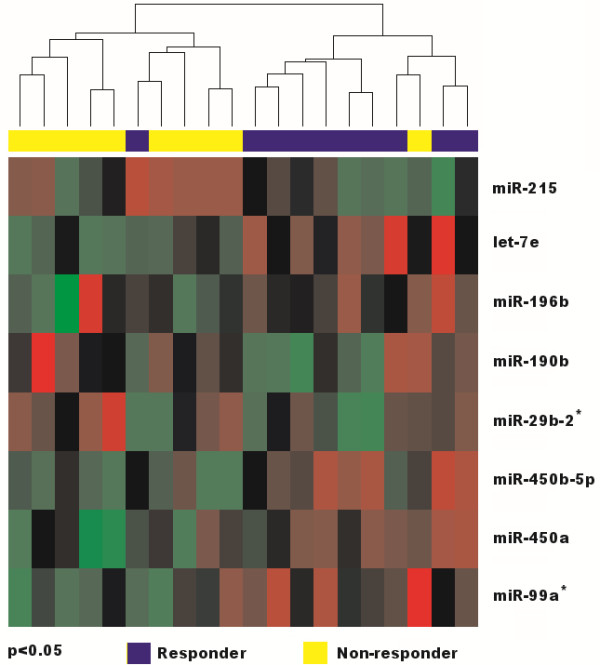
**Hierarchical clustering of 10 responders and 10 non-responders to neoadjuvant chemoradiotherapy stratified according to the expression profile of 8 miRNAs differentially expressed between these two groups.** Yellow color indicate samples of non-responders, blue responders to neoadjuvant therapy, p<0.05.

To determine whether the miRNA expression profiles differ between responders and non-responders to the neoadjuvant chemoradiotherapy in rectal cancer patients, large-scale miRNA expression analysis was performed on 20 samples of preoperative biopsies of rectal cancer tissues (see Table 1 for Patients characteristics). Through LIMMA approach 8 miRNAs indicating significantly different expression levels between both groups were identified (see Table 2). Of these, miR-215, miR-190b and miR-29b-2 have been overexpressed, and let-7e, miR-196b, miR-450a, miR-450b-5p and miR-99a have shown lower expression levels in non-responders. Using these miRNAs, we were able to correctly classify 9 of 10 (90%) responders and 9 of 10 (90%) non-responders (p < 0.05). The results are graphically presented as hierarchical clustergram in Figure 1.

**Table 2:** Overview of miRNAs with significantly different levels of expression in rectal tumors of responders and non-responders to neoadjuvant chemoradiotherapy

**MiRNAs**	**R**	**NR**	**Fold change (NR vs R)**	**P-value**	**Putative targets**^**†#**^
**miR-450b-5p**	U	D	0.07	0.0003	RANBP9, SLC19A2, XIAP, RGMB, SMAD2, SERPINA5, SOX2, TCF5, TIMP2, TGFBR2
**let-7e**	U	D	0.48	0.0075	NRAS^**#**^, KRAS^**#**^, SOCS1^**#**^, HMGA2^**#**^, ABCC5, HOXD1, MASP1, ERCC6, IGF1
**miR-450a**	U	D	0.16	0,0104	MAP3K2, RAB31, TOPBP1, CREB1, DNMT3A, EGFR, ERCC5
miR-99a*	U	D	0,21	0,0163	RAD51C, RAD9B, TIAM1, TMEM87a, TMEM71, SOCS4, RANBP4, RANBP6
**miR-190b**	D	U	4.03	0.0290	CDKN1B, MUC17, MYCBP2, SMAD2, TCF4, CASP2, TP53INP1
miR-29b-2*	D	U	4.25	0.0375	AKT3, RANBP9, PARP2, HDAC5, CDKN3, AGR2, SLC19A2, FOXN3
**miR-215**	D	U	4.40	0.040	ZEB2^**#**^, ALCAM^**#**^, TYMS (TS) ^**#**^, DHFR^**#**^, EREG, HOXB9, NOD2
**miR-196b**	U	D	0.42	0,043	HOXB8^**#**^, HOXC8^**#**^, ERG^**#**^, BACH1^**#**^, FAS, TBRG1, TOX3

Legend: R – responders, NR – non-responders, D – down-regulated expression, U – up-regulated expression. ^**#**^ Target is experimentally validated. ^**†**^ Putative targets were predicted using miRWalk database and miRanda algorith (21) and subsequently selected on the basis of their significance in tumor biology, particularly in the processes of cell survival and resistance to anti-cancer treatment.

## Discussion

Approximately 20% of LARA’s are primary resistant tumors in which there is no reduction or just minimal reduction in the number of cancer cells after neoadjuvant therapy based on concurrent application of radiation and chemotherapy with fluoropyrimidines [14-16]. Should there be valid predictive factors, these patients would be spared exposure to chemotherapy and radiation associated with substantial adverse effects and costs and surgery could be scheduled without delay.

MiRNAs constitute a robust regulatory network with post-transcription regulatory efficiency for almost one half of human coding genes, including important oncogenes, tumor suppressor genes, and genes associated with the invasion, dissemination, and therapy resistance of many tumors [17]. We have used a large-scale miRNA expression profiling and identified 8-miRNAs signature (miR-215, let-7e, miR-196b, miR-190b, miR-29b-2*, miR-450a, miR-450b-5p and miR-99a*) enabling correct classification of 90% of responders (9/10) and non-responders (9/10) to neoadjuvant chemoradiotherapy in patients with LARA. In 5 (miR-215, miR-99a*, miR-196b, miR-450b-5p and let-7e) out of 8 identified miRNAs a potential linkage was already established between their expression and radioresistance or chemoresistance to thymidylate synthetase (TS) inhibitors.

The most frequently studied and the most promising miRNA identified in our study is miR-215, because current knowledge partly enables mechanistical explanation of its association with chemoradioresistance. Song et al. provides direct evidence that miR-215 regulates the TS mRNA in HCT116 colon cancer cell line. Ectopic expression of miR-215 decreased the expression of TS mRNA and protein and at the same time miR-215 inhibits cell proliferation and increased chemoresistance to TS inhibitor raltitrexed. Inhibition of cell proliferation and subsequent chemoresistance was caused by the induction of G2-arrest [22]. Similar results were published by Boni et al., who used in their experiments 5-fluorouracil [23]. Cell cycle arrest as a result of increased expression of miR-215 was confirmed on model of colorectal cancer also by another independent study [24] and our previous results [25]. A further and very important discovery was the fact that miR-215 regulates the cell cycle not only in colon cancer cells but also in colon cancer stem cells. Recent data suggest that colon cancer stem cells may utilize miR-215 to slow cell proliferation and avoid damage caused by chemotherapy and radiotherapy until receiving a proliferation and differentiation signal [22].

Observations on *in vitro* models were confirmed in clinical practice as well. Karaayvaz et al. showed that high levels of miR-215 expression in cancer tissues are closely associated with poor overall survival of patients with colon cancer in stage II and III (HR 3.516; P=0.025) [26]. These results are in agreement with our results showing down-regulation of miR-215 in LARA responding to chemoradiotherapy.

The let-7 family of miRNAs (let-7a through let-7h) regulates expression of key oncogenes, such as RAS and MYC, and is specifically down-regulated in many cancer types. Weidhaas et al., reported that the let-7 family is over-represented in a class of miRNAs exhibiting altered expression in response to radiation. Using *C. elegans*–based *in vivo* model of radiation-induced reproductive cell death, they confirm the ability of let-7 family of miRNAs to increase radiosensitivity when over-expresed [27]. Accordingly, we have observed up-regulation of let-7e in tumors of therapy responders.

Another predictive miRNA identified in our study, miR-99a*, was previously observed in work of Bandres et al. as up-regulated in tumors of responders to neoadjuvant chemoradiotherapy [28]. Interestingly, among putative targets of miR-99a* are important proteins involved in DNA repair – RAD51C and RAD9B [29]. Up-regulation of miR-99a* in tumors could be associated with lower DNA repair capacity through down-regulation of these genes, which may lead to radiotherapy sensitization. One of the putative targets of miR-450b-5p, another up-regulated miRNA in tumors of patients responding to therapy, is a gene coding the X-linked inhibitor of apoptosis protein (XIAP). It was shown that knockdown of XIAP *in vitro* lead to sensitization of colon cancer cells to irradiation [30]. In case of miR-196b, our research group published a study in which we established that elevated expression of miR-196b was positively correlated with overall survival (HR 0.5470; P = 0.0492) in glioblastoma patients treated with concomitant chemoradiotherapy [31].

Till now, there are only two studies that analyzed global miRNA expression profiles in LARA to find potential predictive miRNAs for response to neoadjuvant chemoradiotherapy. The first study was performed by Scarpati et al. [18]. They have found 14 miRNAs (miR-1183, miR-483-5p, miR-622, miR-125a-3p, miR-1224-5p, miR-188e5p, miR-1471, miR-671-5p, miR-1909, miR-630, miR-765, miR-1274b, miR-720, hsv1-miR-H1) differentially expressed between group of tumors in which, after neoadjuvant chemoradiotherapy, a complete remission occured (TRG1/pCR) vs. control group that contained all other tumors (TRG2, TRG3, TRG4). There is no overlap between miRNA signature identified in this study and our results. We suppose that the cause of this discrepancy is in a different and, in part, also unsuitable design of Scarpati’s study, as 55% of tumors in control group reached expressive regression of cancer cells classified by TRG2 grade. This evaluation practically means that there was almost complete remission of tumor cells. Moreover, patients in their study received only dose of 45 Gy and as chemotherapy oxaliplatine was used. From biological point of view, it can be expected that tumors in which total or almost total regression was achieved after neoadjuvant chemoradiation therapy, would share same or similar gene profiles and protein expression. It can also be expected that if tumors in Scarpati study were given radiation dose of 50 Gy, most of the tumors in group TRG2 would reach total eradication of tumor cells (TRG1/pCR).

Second study was published in the form of an abstract by Bandres et al. on ASCO 2012 Annual Meeting (28). Bandres’ and our studies are much more similar since we used the same therapy procedures for patients with LARA and we also used similar designs for the study. Bandres et al. identified a miRNA signature that correctly differentiated extreme-phenotype of responders (TRG1) and non-responders (TRG4). They found up-regulation of miR-21*, miR-99*, miR-125b, miR-125b1*, let-7c and miR-490 to be significantly correlated with a higher likelihood of achieving TRG1/TRG2 response, and down-regulation of miR-21* and miR-125a-3p to be associated with a TRG-4 response. Our results are in agreement at least in case of miR-99* and let-7 family. As far as the rest of miRNAs is concerned, only in case of miR-21* our data showed a difference between group of responders and non-reponders indicating trend but have not reached statistical significance (p=0,11).

## Conclusion

Taken together, our results support hypothesis that miRNAs are part of the mechanisms that are involved in response of rectal cancer to the chemoradiotherapy and that miRNA’s could be promising predictive biomarkers for patients undergoing such treatment. In most of miRNA’s we identified (miR-215, miR-99a*, miR-196b, miR-450b-5p and let-7e), the potential linkage between their expression and radioresistance or chemoresistance to inhibitors of thymidylate synthetase was already established, but some of them were identified for the first time. Therefore, the mechanisms and predictions obtained in this study need to be further validated in more detailed models and on an independent set of patients, before applying in clinical practice.

## Competing interests

There is no conflict of interest to report for this article.

## Authors’ contributions

MS, PF, IK, JG and RV collected the RNA samples and clinical data of patients and controls involved in the study. MS and OS designed the study, performed analysis and interpretation of data, and critical revision of the manuscript. MS, JS and OS participated in the manuscript preparation. JS, JN and OS performed the RNA purification and miRNA expression profiles analysis. LR performed statistical evaluation of the data. All authors read and approved the final manuscript.
